# The effectiveness of depression management for improving HIV care outcomes in Malawi: protocol for a quasi-experimental study

**DOI:** 10.1186/s12889-019-7132-3

**Published:** 2019-06-26

**Authors:** Michael Udedi, Melissa A. Stockton, Kazione Kulisewa, Mina C. Hosseinipour, Bradley N. Gaynes, Steven M. Mphonda, Brian W. Pence

**Affiliations:** 1 NCDs and Mental Health Unit, Ministry of Health, P. O. Box 30377, Capital City, Lilongwe 3, Malawi; 2 Department of Epidemiology, University of North Carolina at Chapel Hill, Gillings School of Global Public Health, 135 Dauer Dr, Chapel Hill, NC 27599 USA; 3 Department of Mental Health, University of Malawi, College of Medicine, P/Bag 360, Chichiri, Blantyre 3, Malawi; 4 Tidziwe Centre, University of North Carolina Project-Malawi, Private Bag A-104, Lilongwe, Malawi; 5 Department of Psychiatry, University of North Carolina at Chapel Hill School of Medicine, 333 S Columbia St, Chapel Hill, NC 27516 USA

**Keywords:** HIV/AIDS, Mental health, Depression, Sub-Saharan Africa, Malawi, Integration, Task-shifting, Program evaluation, Implementation science, Retention

## Abstract

**Background:**

Depression, prevalent among people living with HIV (PLWH) in Malawi, is associated with negative HIV patient outcomes and likely affects HIV medical management. Despite the high prevalence of depression, its management has not been integrated into HIV care in Malawi or most low-income countries.

**Methods:**

This study employs a pre-post design in two HIV clinics in Lilongwe, Malawi, to evaluate the effect of integrating depression management into routine HIV care on both mental health and HIV outcomes. Using a multiple baseline design, this study is examining mental health and HIV outcome data of adult (≥18 years) patients newly initiating ART who also have depression, comparing those entering care before and after the integration of depression screening and treatment into HIV care. The study is also collecting cost information to estimate the cost-effectiveness of the program in improving rates of depression remission and HIV treatment engagement and success.

**Discussion:**

We anticipate that the study will generate evidence on the effect of depression management on HIV outcomes and the feasibility of integrating depression management into existing HIV care clinics. The results of the study will inform practice and policy decisions on integration of depression management in HIV care clinics in Malawi and related settings, and will help design a next-step strategy to scale-up integration to a larger scale.

**Trial registration:**

ClinicalTrials.gov ID [NCT03555669]. Retrospectively registered on 13 June 2018.

## Background

The Joint United Nations Programme on HIV/AIDS (UNAIDS) “90–90-90” goals provide a compelling roadmap toward achieving the end of the HIV epidemic. The UNAIDS plan, which has been embraced by the international public health community and much of sub-Saharan Africa, calls for 90% of those HIV-infected to be aware of their status, 90% of those aware of their status to be on sustained antiretroviral therapy (ART), and 90% of those on ART to be virally suppressed [1]. Achievement of these goals is expected to dramatically reduce or end the HIV epidemic [1, 2]. In parts of sub-Saharan Africa (SSA), early retention in HIV care is a major obstacle to achieving the UNAIDS 90–90-90 goals. While adherence and viral suppression among those remaining in care is high [3], initiation of and retention in ART treatment (the “second 90”) has been challenging. The first year of ART treatment is a particularly vulnerable period: nearly a quarter of people initiating ART are lost to care within the first 12 months, with the majority of loss to care after the first visit or within the first few months of care [4–6].

Comorbid depression renders people living with HIV vulnerable. Comorbid depression affects patients receiving ART [7] as it is a barrier to ART retention [8, 9], associated with reduced ART adherence and viral suppression [7, 10–12].

Malawi, with a population of 17.5 million, has an adult HIV prevalence of 10% [13] and barriers reflecting challenges experienced elsewhere in sub-Saharan Africa. The Malawi Ministry of Health (MoH) has embraced the UNAIDS plans and has been a leader and innovator in expansion of antiretroviral therapy (ART) treatment programs. Yet retention in HIV care has been challenging, with only 76% of adults initiating ART being retained in care at 12 months, and only 65% being both retained in care and virally suppressed [14]. Depression is prevalent among HIV-infected adults in Malawi [15, 16] and is associated with decreased retention in care and viral suppression [7–12, 17–20]. However, Malawi has few psychiatrists and psychiatric wards to provide care [21]. The MoH is working to build capacity through task-sharing approaches, including training primary care providers and outreach workers in mental health screening and counselling [22–24].

Depression is a prime target for strategies to improved HIV outcomes in Malawi. Depression treatment has been linked to improved ART adherence in a meta-analysis comparing those receiving vs. not receiving depression treatment [25], a conclusion consistent with many observational studies [20, 26, 27], although evidence from intervention studies is mixed [28–33]. Preliminary evidence suggests that depression care may improve HIV outcomes in Africa [34–38]. In a pilot study of the integration of antidepressant management into HIV clinical care in Cameroon, in which patients with HIV and depression received treatment of amitriptyline, 87% of patients achieved full remission of their depression within 3 months [37]. Furthermore ARV adherence, viral suppression, and self-reported health also improved. Although the Cameroon study was a single-arm pilot with no comparison group, it suggests that appropriate depression care for people initiating ART may be important to achieve the 90–90-90 goals. However, more robust evidence of the impact of depression treatment on HIV care retention and treatment outcomes will be critical to help guide the allocation of resources to optimize HIV treatment outcomes.

The purpose of this paper is to present the protocol for a quasi-experimental study designed to estimate the effect of the integration of depression treatment into routine HIV primary care on both mental health and HIV-related outcomes in Malawi.

## Methods/design

### Purpose

Our evaluation study has two objectives. The primary objective of this evaluation is to assess the impact of a pragmatic, scalable mental health treatment program on HIV care outcomes including retention in care and viral suppression. The secondary objective of this study is to evaluate the impact of the mental health treatment program on mental health outcomes, including depression remission and depression response.

Specifically, our study is designed to evaluate the following hypotheses:

#### Primary

**H1** Compared to the period prior to the depression treatment program, adults with HIV and depression during the implementation of the treatment program will be more likely to be retained in HIV care and virally suppressed 6 months after ART initiation (**primary outcome**).

#### Secondary

**H2** Compared to the period prior to the depression treatment program, adults with HIV and depression during the implementation of the treatment program will be more likely to be virally suppressed 6 months after ART initiation.

**H3** Compared to the period prior to the depression treatment program, adults with HIV and depression during the implementation of the treatment program will be more likely to be retained in HIV care 6 months after ART initiation.

**H4** Compared to the period prior to the depression treatment program, adults with HIV and depression during the implementation of the treatment program will have higher HIV care appointment adherence (proportion of scheduled visits that were attended) over the first 6 months on ART.

**H5** Compared to the period prior to the depression treatment program, adults with HIV and depression during the implementation of the treatment program will be more likely to have achieved depression remission 6 months after ART initiation.

### Study design

**Fig. 1:**
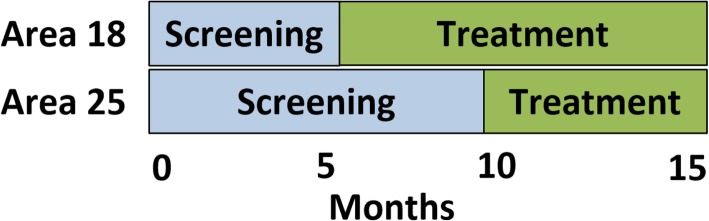
Program implementation Phases

The study employs a multiple baseline evaluation design in two clinics to evaluate the impact of the integrated depression treatment program on HIV outcomes (Fig. 1). The reasons for the choice of the study design has been described published elsewhere [39].

Study activities will last 27 months. This period includes abstracting depression screening data on patients starting ART over a 20-month period and abstracting additional treatment and outcome data for an additional 7 months to identify follow-up viral load, appointment attendance, and depressive symptom outcomes. An additional 3 months is anticipated to complete data abstraction, analyses and dissemination activities.

### Measures

We are abstracting data on HIV and mental health outcomes for patients newly initiating ART who screen positive for depression (Table 1).

**Table 1:** Measures

	Measure	Source	Time Point
Viral Load	viral load copies/mL	Mastercard or EMR	6 month
Appointment Attendance	Appointment Dates	Mastercard or EMR	Monthly
Depressive severity	PHQ-9 Scores	Mastercard	Monthly

*EMR* Electronic Medical Record

Depressive severity is being measured using the Chichewa version (vernacular language for Malawi) of the Patient Health Questionnaire (PHQ)-9. The score of 5–9 on the PHQ-9 is considered mild depression while that of 10 and above is moderate to severe depression [40]. Providers screen all patients for depression at the time of ART initiation by administering the PHQ-9 rather than self administration because of the low literacy levels among the patients. Patients who score ≥ 5 at ART initiation are re-assessed with the PHQ-9 by the HIV provider at each subsequent ART visit. This information is captured on a form called the Mental Health Mastercard that is kept with the patient’s HIV file.

Depression Remission will be defined as a PHQ-9 score < 5 at 6 months.

Retention in HIV care will be defined as being “on time” to all scheduled ART return visits in the first 6 months on treatment. “On time” will be defined as no more than 30 days late; a secondary, tighter definition will define “on time” as no more than 14 days late At ART initiation and each subsequent ART appointment, providers give patients a follow-up appointment date and a sufficient supply of ART. This information is recorded in the patient’s chart, called an HIV Mastercard, and in the Electronic Medical Record (EMR). However, generally for the first 6 months of care, new ART patients receive a 30-day supply of ART and a return appointment date in 30 days. Most often, patients need to attend monthly ART refill appointments for their first 6 months of care in order to maintain ART supply through 6 months.

Consistent ART supply will be defined as never going more than 5 days without ART in the first 6 months, calculated from the days’ supply of ART dispensed at each appointment in the first 6 months and the time between appointments.

Viral Suppression will be defined as a viral load < 1000 copies/mL at 6 months. A viral load threshold of 1000 copies/ml was chosen as this is the threshold used in the resource-limited Malawian health care system to guide decisions about treatment failure and second-line alternatives. Viral load testing is performed at Bwaila Hospital in Lilongwe using Abbott m2000 RealTime HIV-1 assay instruments with a lower limit of detection of 40 copies/mL. The results of the viral load test are recorded in the EMR.

### Intervention program

We integrated depression screening and treatment into ART care into two clinics in Lilongwe, Malawi using a multiple baseline design in two phases: a screening phase and a treatment phase.

During the screening phase, HIV care providers screened patients for depression and monitored their depressive symptoms using the Patient Health Questionnaire-9 (PHQ-9). HIV care providers managed patients identified with depression according to existing care pathways, which included: 1) counselling by the HIV care provider; 2) referral to an on-site or off-site psychiatric nurse or other mental health specialist; or 3) in acute cases, transport to the outpatient psychiatric unit at the nearby district hospital.

At the launch of the treatment phase, HIV care providers were trained in algorithm-guided antidepressant management [38]; additionally, clinic-based lay health workers completed training in the Friendship Bench problem-solving therapy counselling protocol [38]. During this phase, providers are continuing to screen patients for depression. The providers manage cases of depression identified during the treatment phase with either antidepressants or problem-solving therapy, and providers monitor their depression treatment response at follow-up visits.

#### Algorithm-based care for depression (ABCD)

We adapted an ABCD antidepressant prescription model. ABCD is a resource-efficient, task-sharing model for delivering high quality, effective, safe antidepressant management in non-psychiatric settings. ABCD equips non-specialists (e.g., primary care clinicians or HIV clinicians) to safely and effectively prescribe and monitor antidepressants. The model trains staff to know what to measure (e.g., depressive severity, side effects, adherence), how and when to measure it, how to interpret the results, and what resulting course of action to pursue (e.g., maintain dose, increase dose, address side effects, switch medication). Key features include (1) treatment decisions guided by regularly measured metrics of depressive severity, side effects, and adherence; (2) an algorithm summarizing best-practices care based on metrics; (3) a treatment goal of remission (full resolution of symptoms); (4) low starting dose followed by dose adjustments until remission, as long as the medication remains tolerable; (5) ensuring an adequate trial (six to eight weeks at moderate to high dose); and (6) regular structured supervision for continuous quality improvement.

**Fig. 2:**
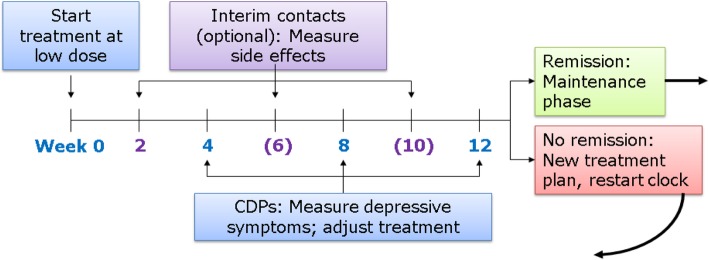
Algorithm-based care for depression (ABCD) Timeline

In our program, a patient initiates an antidepressant when identified with moderate to severe depressive severity (PHQ-9 score ≥ 10) combined with clinical confirmation of the presence of depression (Fig. 2). Every four weeks at the monthly ART appointment, considered critical decision points (CDPs), the patient reports depressive severity and side effects using standardized measures. These metrics guide a recommendation to increase, maintain, or decrease the antidepressant dose or to change treatments. If the patient is tolerating the medication but depressive symptoms have not remitted, a dose increase is recommended. If side effects are not tolerable, the recommendation is to address them or switch treatment. At CDP3 (i.e., after 12 weeks), the patient has either achieved remission (and enters a maintenance phase) or has demonstrated treatment resistance to the current medication (and receives a new treatment plan).

ABCD has proven to be effective in primary care [41, 42] and in HIV care [30, 43] in high-income countries, and more recently has demonstrated safety, feasibility, and acceptability when adapted for HIV care and delivered by a general practice medical providers in Cameroon, Tanzania and Uganda [34, 44, 45].

#### .Friendship bench problem-solving therapy (PST)

We adapted a PST intervention developed for use in Zimbabwe called the Friendship Bench [38]. PST is a psychological treatment that focuses on teaching patients how to identify triggers and effectively manage stressful life events by learning or reactivating problem solving skills [46]. PST evolved from social problem solving theory, framing three sequential phases for addressing problems including (1) discovery (identifying solution), (2) performance (implementing solution), and (3) verification (assessing outcome) [47]. The goal-oriented approach is a cognitive-behavioral intervention focused on improving an individual’s coping abilities through psychoeducation, interactive problem-solving exercises, and developing action plans aimed at reducing psychological distress. PST is easy to learn, has been integrated successfully into primary care settings [48], and is an effective strategy for depression management [49–51].

The original Friendship Bench consisted of task-shifting PST to lay health workers in Zimbabwe; requiring minimal training with structured supervision. The Friendship Bench PST intervention resulted in clinically meaningful improvement in mental health outcomes [38] and was highly acceptable among patients with perceived positive benefits [52, 53]. Other studies from SSA have confirmed the feasibility and effectiveness of PST for management of common mental disorders, including depression [54, 55].

In our program, the developers of the ‘Friendship Bench’ conducted a training for trainers who then went on to train a team of community health workers stationed at each clinic to deliver PST. The program built structures at both clinics for holding Friendship Bench PST sessions. Additionally, we staffed one Friendship Bench counsellor at each clinic throughout the duration of the program.

#### Combined intervention

**Fig. 3:**
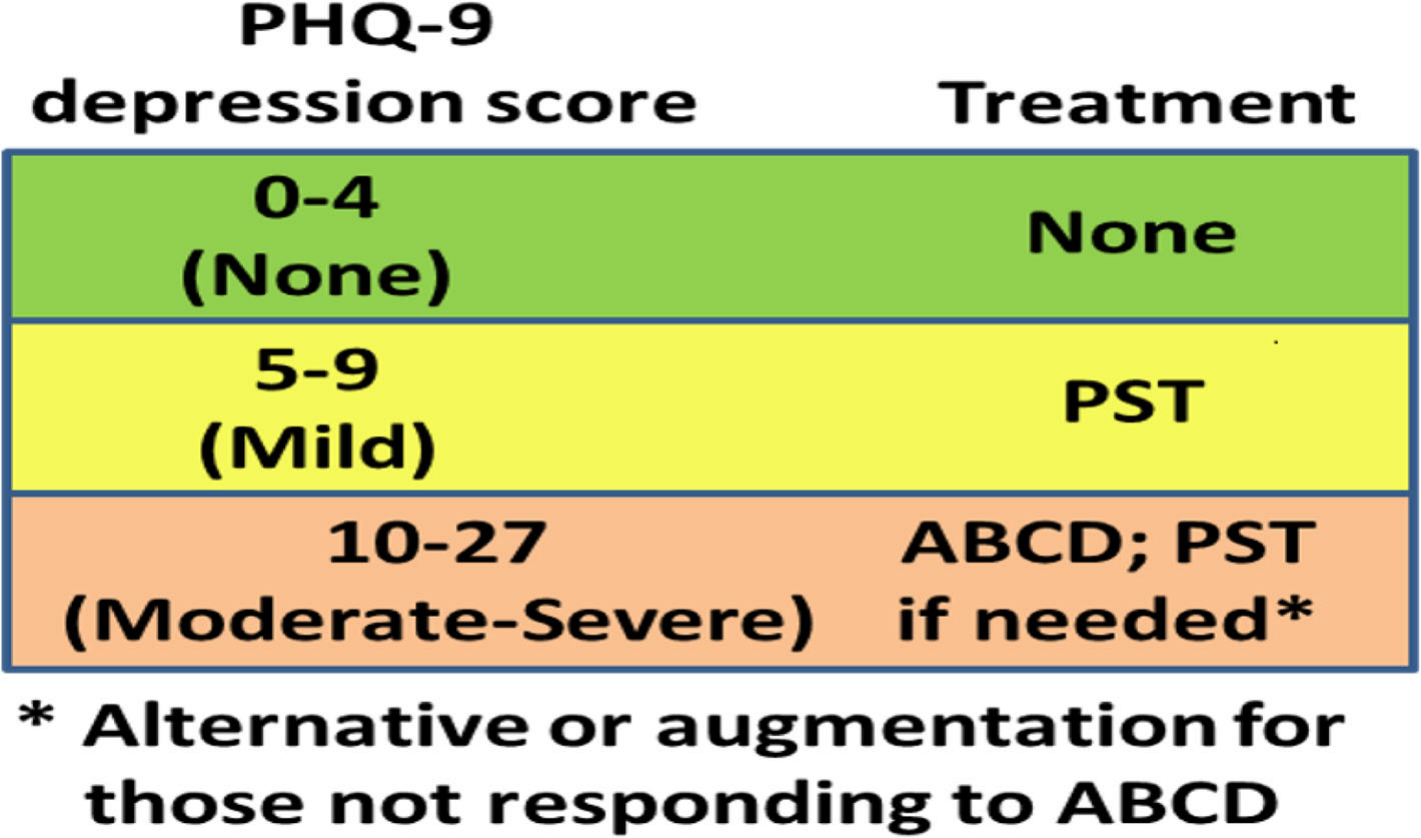
Program Design

Our intervention combined ABCD and PST into a stepped-care intervention with clinical response appropriate to the level of depressive severity (Fig. 3). Patients scoring 0–4 on the PHQ-9, indicating no depression, receive no intervention. Patients scoring 5–9 on the PHQ-9, indicating mild depressive symptoms, receive Friendship Bench PST. Patients scoring ≥10 on the PHQ-9, indicating moderate to severe depressive symptoms, are offered ABCD as the first treatment option, since literature indicates that medications and counselling are equally effective for this group and medication management can be offered more efficiently as first-line within existing clinical care. For this group, Friendship Bench PST is an alternative or augmentation option for those who do not tolerate or do not respond to antidepressant treatment. In all cases, ART providers continue to monitor patients’ depressive severity at follow-up visits with the option of modifying their treatment plan if their symptoms worsen or do not improve after three months of an adequate treatment course.

### Setting

We are conducting the evaluation study at two public-sector primary care HIV clinics in Lilongwe, Malawi: Area 18 and Area 25 health clinics. These clinics are managed by Lilongwe District Health Office. More detailed description of the study setting has been published elsewhere.

### Population and sample size

We are abstracting screening data for all consenting adult (≥18 years old) patients newly initiating ART and screened for depression at the time of ART initiation at the study sites during the study period. Additional depression treatment and outcome data are being abstracted for all patients who screen positive for mild, moderate, or severe depressive symptoms (PHQ-9 total score ≥ 5) [56, 57].

We focused on two populations of interest. For the primary population of interest, all patients with depressive symptoms (PHQ-9 score ≥ 5), the original sample size was determined to provide 80% power to detect an 11 percentage point pre-post difference in the primary outcome. For the secondary (smaller) population of interest, all patients with moderate to severe depression (PHQ-9 score ≥ 10), the original sample size would provide the same power to detect an 18 percentage point pre-post difference. To achieve this power, we would require 645 patients with any depressive symptoms and 225 patients with moderate to severe depressive symptoms.

Based upon the historical rate of new ART patients (122 per month between the two clinics) and the expected prevalence of any depressive symptoms (35%) and moderate to severe depressive symptoms (12%) based on prior literature [58] [7], we originally planned for a 15-month data collection period to accrue the targeted sample. During the course of data collection, the rate of new ART patients was higher than expected but the prevalence of depressive symptoms was lower than expected. After extending the recruitment period for an additional 5 months, the accrual of new ART patients concluded with a final sample of 2084 individuals. The prevalence of mild to severe depression was 24.1% (*n* = 502) and the prevalence of moderate to severe depression was 6.3% (*n* = 131). This sample has 80% power to detect a slightly larger difference in the primary outcome of 12 percentage points (e.g. 60% vs. 72%) or a risk ratio of 1.2 for those with mild to severe depression. For those with moderate to severe depression, the sample has 80% power to detect an improvement in the primary outcome of 22 percentage points (e.g. 60% vs. 82%) or a risk ratio of 1.4.

### Data abstraction and management

Data on viral loads, HIV appointment attendance, and depressive symptoms is abstracted from electronic and paper medical records and the depression treatment registry at the sites. ART appointment data including the date of the appointment, the expected return date, and medication prescribed is recorded in the EMR, on a paper patient file (called an HIV Mastercard), or both. The results of the viral load tests are recorded in the EMR. Data on patients’ depressive symptoms and depression treatment are only recorded on paper in an additional patient filed attached to the ART mastercard (called a mental health mastercard). The community health workers responsible for administering the Friendship Bench problem-solving therapy also maintain paper records (called a Counselling Mastercard) that capture engagement with the PST sessions. Cost information was collected using a time-audit approach to estimate costs of delivering the program, including the opportunity cost of training participation and the time providers and supervisors spend to deliver and supervise the program. Time-audit data was captured through a combination of direct observation of medical provider and counsellor encounters supplemented with self-reporting by counsellors.

### Data analysis

The primary program evaluation question is whether the depression treatment program results in improved HIV and mental health outcomes for patients with HIV and depression relative to the standard of care prior to program implementation. The main analysis will follow an “intent-to-treat” approach, classifying patients according to screening vs. treatment phase (unexposed vs. exposed to the program) without regard to actual treatment received. In our main analysis, we will define our primary outcome, retention in HIV care with viral suppression 6 months after starting ART, as never being more than 30 days late to any scheduled return visit in the first 6 months on ART and having a viral load < 1000 copies/mL at 6 months; patients not retained or not suppressed will be coded as a failure. (Table 2**)** This composite outcome was chosen as the primary outcome because it is the definition of ART clinical success in the Malawian ART treatment system; those not retained and those retained but not suppressed are both clinical failures. This main analysis will estimate the probability of retention and viral suppression 6 months after starting ART using a generalized linear model with binomial error distribution, adjusting for calendar time and potential confounders that are imbalanced between arms at baseline, potentially including age, sex, clinic, PHQ-9 score, and presence of suicidal ideation. We will model these associations using separate generalized linear models among two samples: (a) those with mild, moderate, or severe depression (PHQ-9 ≥ 5) and (b) those with moderate to severe depression (PHQ-9 ≥ 10).

**Table 2:** Outcome Variables

	Definition	Type
Primary Outcome		
Retention with Viral Suppression	both attending all ART appointments “on time” through 6 months and having a viral load < 1000 copies/mL at 6 months	Binary
Secondary Outcomes		
Viral Suppression	viral load < 1000 copies/mL at 6 months	Binary
Retention	attending all ART appointments “on time” through 6 months	Binary
	number of ART appointments attended through 6 months	Count
Consistent ART supply	Never going more then 5 days without ART, as calculated from pills dispensed and time between attended appointments	Binary
Depression Remission	PHQ-9 Score < 5 at 6 months	Binary

In secondary analyses, we will consider ART care retention through 6 months (binary), number of ART appointments attended in the first 6 months (count), maintaining consistent ART supply through 6 months (binary), and viral suppression at 6 months among those retained (binary). Further, secondary analyses will take an “as treated” approach, comparing patients who receive treatment in the treatment phase to all patients during the screening phase; and an “as adequately treated” approach, comparing patients who receive adequate treatment (≥3 consecutive months of protocol-concordant antidepressant prescriptions or ≥ 4 counseling sessions) during the treatment phase to all patients during the screening phase. Data are not available on antidepressant adherence. (Table 3).

**Table 3:** Analyses

Analysis	Definition
Primary: “intent-to-treat”	Initiated after program launch
“as treated”	Received depression treatment
“as adequately treated”	≥3 consecutive months of protocol-concordant antidepressant prescriptions or ≥ 4 counselling sessions

### Timeline

The screening phase began at Clinic A in April 2017 and Clinic B in May 2017. The treatment phase was integrated into Clinic A after 7 months, in November 2017, and into Clinic B after 11 months, in April 2018. Identification of new patients concluded at both clinics in November 2018. Follow-up data abstraction is expected to continue through June 2019.

## Discussion

This novel pilot program is Malawi’s first depression treatment program for people living with HIV and one of the few in the sub-Saharan Africa region to use a task-shifting model to provide both problem-solving therapy and antidepressant depression treatment [34–38]. This study will be the first to evaluate the impact of such a program on both mental health and HIV outcomes and to investigate the potential benefits of this task-shifting depression care model. Findings from this study will directly inform the Malawi Ministry of Health’s ongoing national strategic plans on mental health.

The study design draws both from implementation science and epidemiologic methods, with the ultimate goal of generating evidence-based practices, interventions and policies that can readily (and rapidly) be adopted and integrated into routine care in public sector settings [59–61]. We have conscientiously designed the evaluation to yield high quality, real-world findings on integrating depression management into HIV care that are readily applicable to the implementation of integrated mental health services in non-specialist settings.

The program itself focuses on provider training to manage antidepressants and provide evidence-based counselling, and thus cannot be randomized at the individual level. Since patients do not see the same provider from one appointment to the next, in this context the intervention could not be randomized at the provider level either. A cluster-randomized trial was also beyond the resources available for this project. Given these constraints, a multiple baselines design provides stronger causal inference than would be available with a simple pre-post design. Multiple baseline designs have been advocated as one of the designs providing the strongest causal inference apart from the randomized trial [62].

We anticipate that the study will generate evidence on the effect of depression management on HIV outcomes and the feasibility of integrating depression management into existing HIV care clinics. The results of the study will inform practice and policy decisions on integration of depression management in HIV care clinics in Malawi and similar low-income country settings, allowing design of a scale up strategy.

## Data Availability

The datasets that will be collected and/or analysed over the course of the ongoing study will be available from the corresponding author on reasonable request.

## Abbreviations

ART: Antiretroviral Treatment
EMR: Electronic Medical Record
HIV: Human Immunodeficiency Virus
HTC: HIV Testing and Counseling
IRB: Institutional Review Board
MOH: Ministry of Health
NCDs: Non-communicable Diseases
NHSRC: National Health Science Research Committee
PEPFAR: United States President’s Emergency Plan for AIDS Relief
PHQ-2: Patient Health Questionnaire-2
PHQ-9: Patient Health Questionnaire-9
PST: Problem-solving Therapy
SOAR: Supporting Operational AIDS Research
UNAIDS: Joint United Nations Programme on HIV/AIDS
UNC: University of North Carolina
USAID: United States Agency for International Development
